# Real-World Use of 3rd Line Therapy for Multiple Myeloma in Austria: An Austrian Myeloma Registry (AMR) Analysis of the Therapeutic Landscape and Clinical Outcomes prior to the Use of Next Generation Myeloma Therapeutics

**DOI:** 10.1371/journal.pone.0147381

**Published:** 2016-03-03

**Authors:** Ella Willenbacher, Roman Weger, Ursula Rochau, Uwe Siebert, Wolfgang Willenbacher, Austrian Myeloma Registry (AMR)

**Affiliations:** 1 Medical University of Innsbruck, Internal Medicine V—Hematology and Oncology, Innsbruck, Austria; 2 Area 4 Health Technology Assessment and Bioinformatics, ONCOTYROL–Center for Personalized Cancer Medicine, Innsbruck, Austria; 3 Institute of Public Health, Medical Decision Making and Health Technology Assessment, Department of Public Health, Health Services Research and Health Technology Assessment, UMIT—University for Health Sciences, Medical Informatics and Technology, Hall in Tirol, Austria; 4 Institute for Technology Assessment and Department of Radiology, Massachusetts General Hospital, Harvard Medical School, Boston, Massachusetts, United States of America; 5 Center for Health Decision Science, Department of Health Policy and Management, Harvard T.H. Chan School of Public Health, Boston, Massachusetts, United States of America; Queen's University Belfast, UNITED KINGDOM

## Abstract

**Objective:**

Clinical trials demonstrate improving survival in patients with multiple myeloma (MM) after treatment. However, it is unclear whether increased survival translates to a similar benefit in a real world setting.

**Methods:**

We analyzed the overall survival of 347 multiple myeloma patients in Austria by means of a national registry (AMR), focused on results from 3^rd^ and later lines of therapy. This benchmark was chosen to define a baseline prior to the broad application of upcoming 2^nd^ generation drugs (carfilzomib, pomalidomide).

**Results:**

Projected 10 years survival for patients with MM in Austria is estimated to be 56% in patients diagnosed in between the years 2011–2014, 21% in patients with a diagnosis made between 2000–2005, and 39% in those with a diagnosis made between 2006–2010). For the same intervals a significant increase in the use of both bortezomib, lenalidomide and thalidomide—so called IMiDs (from 2005 onwards) and their simultaneous use in combination therapies (from 2010 onwards) could be shown. The use of autologous transplantation (ASCT) remained more or less constant at ~ 35% of patients in the 1^st^ line setting over the whole period, comparing well to international practice patterns, while the use of 2^nd^ line ASCT increased from 5.5% to 18.7% of patients. Patients in 3^rd^ or later line treatment (n = 105), showed that even in relapsed and refractory disease median survival was 27 months with a considerable proportion of long-term survivors (~20%).

**Conclusion & Perspective:**

With the expected emergence of additional active anti-myeloma compounds, we aim to assess survival in patients with relapsed and refractory MM.

## Introduction

Prognosis and survival of patients suffering from Multiple Myeloma (MM) has been considerably improved within the last decade [1]. This improvement may be mostly due to the introduction of the so called “novel agents" of either the proteasome inhibitor (PI) type (first in-class: bortezomib—BTZ), or the immune modulatory drug-type (IMiDs), like thalidomide (THAL) and lenalidomide (LEN). However, all patients will develop clinical resistance or refractoriness to these compounds, leading to what is known as double refractory MM (DRMM) in which survival is poor (9 months) [2].

No generally accepted standard of therapy has been established to treat these patients and multiple permutations of all active myeloma therapeutics have been used in in treatment of DRMM with no clear advantage for any specific treatment pattern.

With the approval of pomalidomide (POM) by both FDA and EMA, a new benchmark has been set for 3^rd^ line myeloma therapy based on the results of the MM-003 trial [3], while an additional panoply of new myeloma drugs is in late stage clinical development [4], aspiring for registration, or even having accomplished market access, at least in parts of the world (e.g., carfilzomib–CFZ, in the US) [5].

Because an efficacy trial can often overestimate an intervention’s effect when used outside of a clinical trial setting [6]. We decided to the assess real-world evidence for use of all 3^rd^ and later line therapies not comprising POM or CFZ, as documented in the Austrian Myeloma Registry (AMR); www.myeloma.at. On May 21, 2015, the collection and analysis of this type of data was recently been endorsed by the US House Energy & Commerce Committee, voting to pass the latest version of the 21st Century Cures Act [7]. The bill, H.R. 6, released May 19, 2015, encourages greater use of observational studies, patient registries and real-world therapeutic use to support approvals of new drugs and improve post-marketing surveillance.

The aim of our analysis was to describe patient characteristics and outcomes, focusing on overall survival and highlighting confounding factors (e.g. length time bias, lead time bias, patient selection, available therapeutic options, etc.). Furthermore, we aimed to describe the broad range of 3^rd^ line and later line treatment options used in myeloma patients in the AMR.

## Patients & Methods

The AMR was established in 2008 (comprising retrospective inclusion of patients diagnosed back to the year 2000) under the auspices of the Austrian Society for Hematology & Oncology (OeGHO; www.oegho.at) as a web-based quality control project to document all facets of MM treatment in Austria, focusing the rapid proliferation of “*novel agent”* based therapies. Bortezomib became widely available in Austria in May 2005, lenalidomide (for 2^nd^ line use in June 2006), while thalidomide has been available from the late nineties as a magistral formula for individual patients. Additionally, some patients received these drugs earlier as participants in clinical trials, named patient- or early access programs. 16.9% of the patients covered in this analysis received therapy in the setting of controlled clinical trials.

All MM patients in any line of treatment, from the moment of initiation of a registry participant, are available on the basis of written informed consent. The AMR, collecting data from a representative group of MM centers) delivers a comprehensive picture of current national clinical practice and benchmarks actual MM therapy compared to guideline–based recommendations. Participating centers directly benefit from the AMRs evaluation tool, which allows users to analyze data within their own patient populations. This allows to perform freely combinable parameter defined subgroup analyses. To guarantee highest data security standards, the server is located at a neutral hosting partner (OeKB–BS, www.oekb.at) with secured maintenance service (www.asoluto.com). Automated plausibility checks (e.g. for patient doublets) and regular on-site monitoring assure data quality. Documentation of biologic features allows for the search of MM biomarkers and linkage to a myeloma biobank.

All data is stored in a pseudonomized fashion, and all patient records and information are anonymized and de-identified prior to analysis, compatible to national data protection regulations, and the AMR has been approved by all relevant ethical review boards [8]. As of JUN 10^th^ 2014, eleven national and two international myeloma centers actively contribute data to the AMR. (see S1 File). All research projects need a steering committee consent to be performed.

For this study, all data records of Austrian patients with at least three fully documented lines of therapy, not comprising POM or CFZ in 3^rd^ line or later, were included. Treatment lines were defined as proposed by the International Myeloma Working Group (IMWG) [9] (e.g., induction, autologous transplantation, consolidation, and maintenance constitute one treatment line).

All selected data sets were updated by contacting the responsible physician wherever deemed necessary to assure an up-to-date status.

Because the definition of myeloma progression [9] depends mostly on serological parameters registry data are thus consequently biased by different surveillance schedules used at different centers, in contrast to clinical trial settings offering a fixed schedule of assessments to determine progresion. This impedes using parameters such as *progression-free-survival* (PFS), *time-to-progression* (TTP) and *loss-of-remission* (LOR) in an analysis of registry data to avoid lead time and/or length time biases. We therefore focused our analysis on *overall-survival* (OS) and *time-to-next-treatment* (TNT), which are easier to measure objectively, and in our view clinically more important.

## Statistical Analysis

We describe patient characteristics such as gender, age, survival status, and exploratory clinical patient characteristics such as types and number of treatment lines, time to treatment lines, risk factors (e.g., cytogenetics), and overall survival.

Cytogenetic risk stratification was based on FISH results with a positivity cut-off of 10% of positive cells to define the occurrence of an aberration. For comparisons between patients receiving three or more lines of therapies and the entire AMR cohort the abbreviations 3SG and AMR were used. Dichotomous variables are expressed as proportions, continuous variables as means with standard deviations (SD), and survival data were analyzed using the Kaplan-Meier method [10] and estimated 95% confidence intervals (95% CI) for median survival times. In order to make our data comparable to other longitudinal analysis of myeloma treatment outcome, we also report overall survival data stratified by time of diagnosis. For all comparisons, a p-value of < 0.05 was considered statistically significant. To extrapolate shorter-term follow-up data and estimate comparative long-term survival (at 10 years), a Weibull model was fitted to the survival data.

## Results

### Patients & lines of therapies (Fig 1)

**Fig 1:**
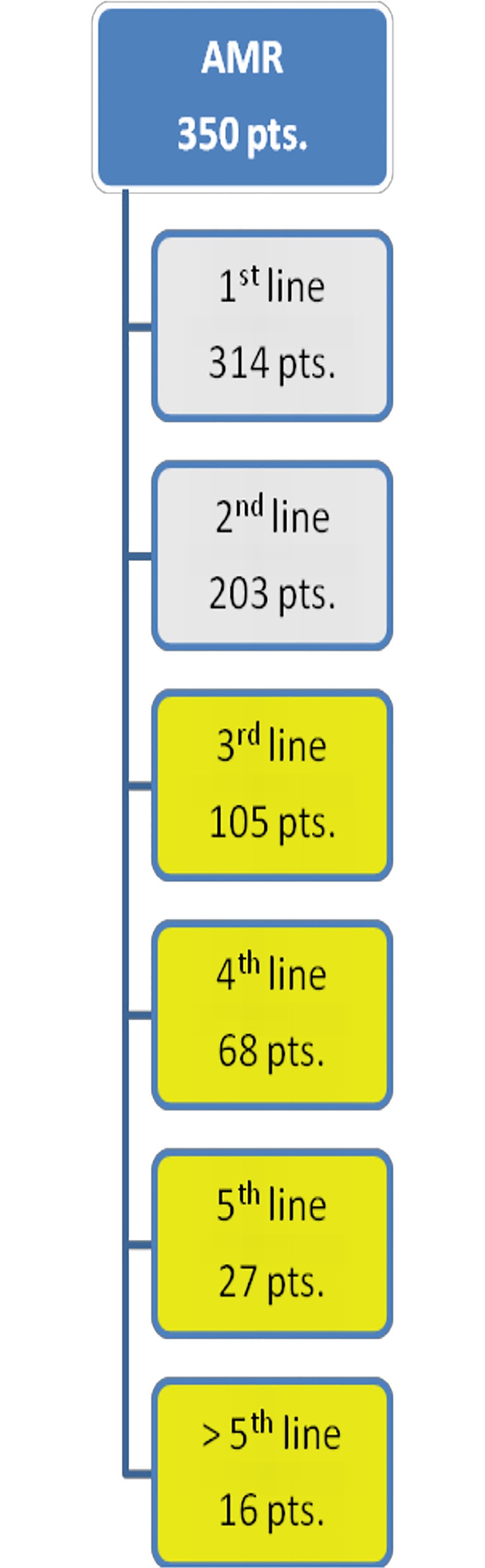
Patients and treatment lines. illustrates the distribution of treatment lines and patients (pts.) documented in the Austrian Myeloma Registry (AMR). Patients who had received at least 3 lines of therapy were included in the analysis (yellow boxes).

As of June 10^th^, 2014, 350 MM patients were documented in the AMR. Out of this cohort, 105 (30%) patients received a 3^rd^ line therapy, while 245 (70%) patients received less than 3 therapeutic regimens. Furthermore, 68 (19.4%) patients received 4^th^ line therapy, 27 (7.7%) received 5^th^ line therapy, and 16 (4.6%) received more than 5 lines of therapy (Fig 1).

The median (SD) time from first diagnosis to start of 3^rd^ line therapy was 39 months (23). The median (SD) follow up of the third line subgroup was 48 months (3.9 months) compared to 24 months (3.6 months) in the entire cohort (Fig 1).

## Patient Disposition (Tables 1 and 2)

There were 193 (56%) males and 154 (44%) females in the overall cohort. In the 3^rd^ line treatment subgroup (3SG) there were 66 (63%) males and 39 (37%) females. Median (SD) age at first diagnosis was 60.5 years (9.05) in males and 65 years (11.22) in females and 61 years (10.05) in all patients. The median (SD) age of the 3SG group was 62.26 years (10.05), with the median (SD) age of male patients being 62.26 years (9.05) and 62.83 years (11.22) in females. In the AMR cohort, 182 (52%) patients were alive at the time of analysis and 157 (45%) were deceased. Corresponding figures in the 3SG group were 70 (67%) of the patients deceased and 35 (33%) still alive at the time of analysis.

**Table 1:** Patient disposition in patients receiving or not receiving 3^rd^ line therapy for Multiple myeloma in Austria.

	ENTIRE AUSTRIAN MYELOMA REGISTRY COHORT	SUBGROUP RECEIVING A 3^rd^ and/or HIGHER LINE THERAPY
	AMR^a^	3SG^b^
**N (%)**	350 (100)	105 (100)
**Sex male (%)**	193 (56)	66 (63)
**Sex female (%)**	154 (44)	39 (37)
**Age +/- SD**^c^ **at first diagnosis all pts.**	63.86 a +/- 11.82 a	62.26 a +/- 10.05
**Age +/- SD**^c^ **at first diagnosis male pts.**	62.83 a +/- 11.41 a	62.83 a +/- 11,22 a
**Age +/- SD**^c^ **at first diagnosis female pts.**	65.14 a +/- 12.24 a	61.79 a +/- 9.05 a
**Median follow up**	24 months +/- 3.6 months	48 months +/- 3.9 months
**Alive (%)**	182 (52)	35 (33)
**Dead (%)**	157 (45)*	70 (67)
**Median treatment lines (range)**	2 (1–12)	3 (3–12)
**1**^**st**^ **line therapy ASCT**^e^ **(%)**	77 (22)	21 (20)
**1**^**st**^ **line therapy BTZ**^f^ **based (%)**	167 (48)	52 (49)
**1**^**st**^ **line therapy IMiD**^g^ **based (%)**	87 (33)	25 (31)
**1**^**st**^ **line therapy BTZ & IMiD based (%)**	52 (13)	15 (12)
**2**^**nd**^ **line therapy ASCT**^e^ **(%)**	30 (9)	11 (10)
**2**^nd^ **line therapy BTZ**^f^ **based (%)**	62 (48)	39 (50)
**2**^**nd**^ **line therapy IMiD**^g^ **based (%)**	70 (54)	25 (31)
**2**^**nd**^**line therapy BTZ & IMiD based (%)**	6 (5)	2 (1)
**Cytogenetics informative (%)**	252 (73)	71 (68)
**Cytogenetics non informative (%)**	75 (22)	26 (25)
**Cytogenetic very high risk del17p**^h^	24 (7)	10 (10)
**Cytogenetic very high risk t(4;14)**^i^	8 (2)	4 (4)
**Cytogenetic very high risk both del17p & t(4;14)**	3 (1)	0 (0)
**TNT**^k^ **1**^**st**^ **to 2**^**nd**^ **line therapy**	12.0 months	16.7 months, n.s. (p = 0.277)
**TNT**^k^ **2**^**nd**^ **to 3**^**rd**^ **line therapy**	8.7 months	12.5 months, n.s. (p = 0.198)

^a^ AMR: Austrian Myeloma Registry

^b^ 3SG: 3^rd^ line therapy subgroup

^c^ SD: standard deviation

^d^ pts.: patients

^e^ ASCT: Autologous stem cell transplantation

^f^ BTZ: bortezomib

^g^ IMIiD: immune-modulatory-drug

^h^ del17p: deletion17p

^I^ t(4;14): translocation (4;14)

^j^ mo.: months, a: years

^k^ TNT: time to next treatment

*—difference due to “lost to follow up”, therapies sum up to > 100% due to combination therapies, n.s.: not significant

**Table 2:** Patient disposition in Multiple Myeloma patients diagnosed 2000–2005, 2006–2010 and 2011–2014.

Diagnosis	2000–2005	2006–2010	2011–2014
N (%)	73 (100.0)	136 (100.0)	91 (100.0)
Sex male (%)	39 (53.4)	85 (62.5)	51 (56.0)
Sex female (%)	34 (46.6)	51 (47.5)	40 (44.0)
Age +/- SD^c^ at first diagnosis all pts.	62 a (+/- 11.7 a)	63 a (+/- 12. 4 a)	68 a (+/- 12.1 a)
Alive (%)	13 (17.8)	86 (63.2)	74 (81.3)
Dead (%)	60 (82.2)	50 (36.8)	17 (18.7)
Median treatment lines (range)	2 (1–5)	2 (1–6)	3 (1–12)
1^st^ line therapy ASCT^b^ (%)	22 (30.1)	50 (37.5)	34 (37.4)
1^st^ line therapy BTZ^C^ based (%)	2 (2.7)	13 (9.6)*	31 (34.1)
1^st^ line therapy IMiD^d^ based (%)	40 (54.8)	30 (22.1)*	3 (3.3)
1^st^ line therapy BTZ & IMiD based (%)	3 (4.1)	52 (38.2)*	62 (68.1)*
1^st^ line therapy other (%)	24 (32.9)	1 (0.7)*	7 (7.7)
2^nd^ line therapy ASCT^b^ (%)	4 (5.5)	18 (13.2)*	17 (18.7)
2^nd^ line therapy BTZ^c^ based (%)	0 (0.0)	21 (15.4)*	23 (25.3)
2^nd^ line therapy IMiD^d^ based (%)	16 (21.9)	44 (32.4)*	17 (18.7)
2^nd^line therapy BTZ & IMiD based (%)	8 (11.0)	30 (22.1)*	18 (19.8)
2^nd^line therapy other (%)	12 (16.4)	7 (5.1)	10 (11.0)
Cytogenetic very high risk (any)	6 (8.2)	14 (10.3)	12 (18.7)
Cytogenetic very high risk del17p^e^	6 (8.2)	9 (6.6)	9 (9.9)
Cytogenetic very high risk t(4;14)^f^	0 (0)	5 (3.7)	3 (3.3)
Cytogenetic very high risk both del17p & t(4;14)	0 (0)	2 (1.5)	1 (1.1)

^a^ N = number

^b^ ASCT = autologous stem vell transplantation

^c^ BTZ = Bortezomib

^d^ = Immunomodulatrory Drug (lenalidomide, thalidomide)

^e^ del17p = deletion 17p

^f^ t(4;14) = translocation t(4;14)

* significant changes

Autologous transplantation (ASCT) comprised 30% of 1^st^ or 2^nd^ line treatments for those in the 3SG group (21% in 1^st^ line, 11% in 2^nd^ line) and 31% of 1^st^ or 2^nd^ line treatments in the AMR cohort (22% in 1^st^ line, 9% in 2^nd^ line). A detailed analysis of transplant use over time shows an increase of combined 1^st^ and 2^nd^ line ASCT rates from 35.6% (between 2000–2005), to 50.7% (between 2011–2014).

There were 167 (48%) in the AMR cohort and 52 (49%) of patients in the 3SG group who received a BTZ-based 1^st^ line therapy, 87 (33%) in the AMR cohort and 25 (31%) in the 3SG group who received an IMiD based 1^st^ line therapy and 52 (13%) and 15 (12%) patients who received both an IMiD and BTZ in their first line therapies in the AMR and 3SG groups, respectively.

There were 62 (48%) in the AMR cohort and 39 (49%) in the 3SG group who received a BTZ-based 2^nd^ line therapy, 70 (54%) in the AMR cohort and 25 (31%) in the 3SG group who received an IMiD based 2^nd^ line therapy and 6 (5%) 2 (1%) received both an IMiD and BTZ in their 2^nd^ line therapies in the AMR cohort and 3SG group, respectively.

There were highly significant changes to the use of *novel agents* over time (see Table 2) where an increase in the use of both bortezomib, lenalidomide and thalidomide, (from 2005 onwards) and their simultaneous use in combination therapies (from 2010 onwards) could be observed.

Maintenance and consolidation strategies were used only rarely in both groups and thus not suitable to analysis. No significant differences were seen in treatment patterns of patients receiving a third or -higher line of therapy and those that did not.

Cytogenetic very high risk features [11](del 17p, t(4;14), or both) were present in 14% of 3SG patients as compared to 10% in the AMR total population.

Time-to-next Treatment (TNT) from 1^st^ to 2^nd^ line was 16.7 +/- 5 mo. in the 3SG patients and 12.5 +/- 9 mo. for all patients TNT from 2^nd^ line to 3^rd^ line was found to be 12.5 months (3SG) and 8.7 months (AMR) respectively.

## Survival of MM Patients in Austria 1990–2014 (Fig 2)

Overall survival (OS) from start of 1^st^ line therapy was 70% at 4 years for the entire AMR cohort, compared to 48 months (+/- 26.4 months) for the 3^rd^ line therapy subgroup.

**Fig 2:**
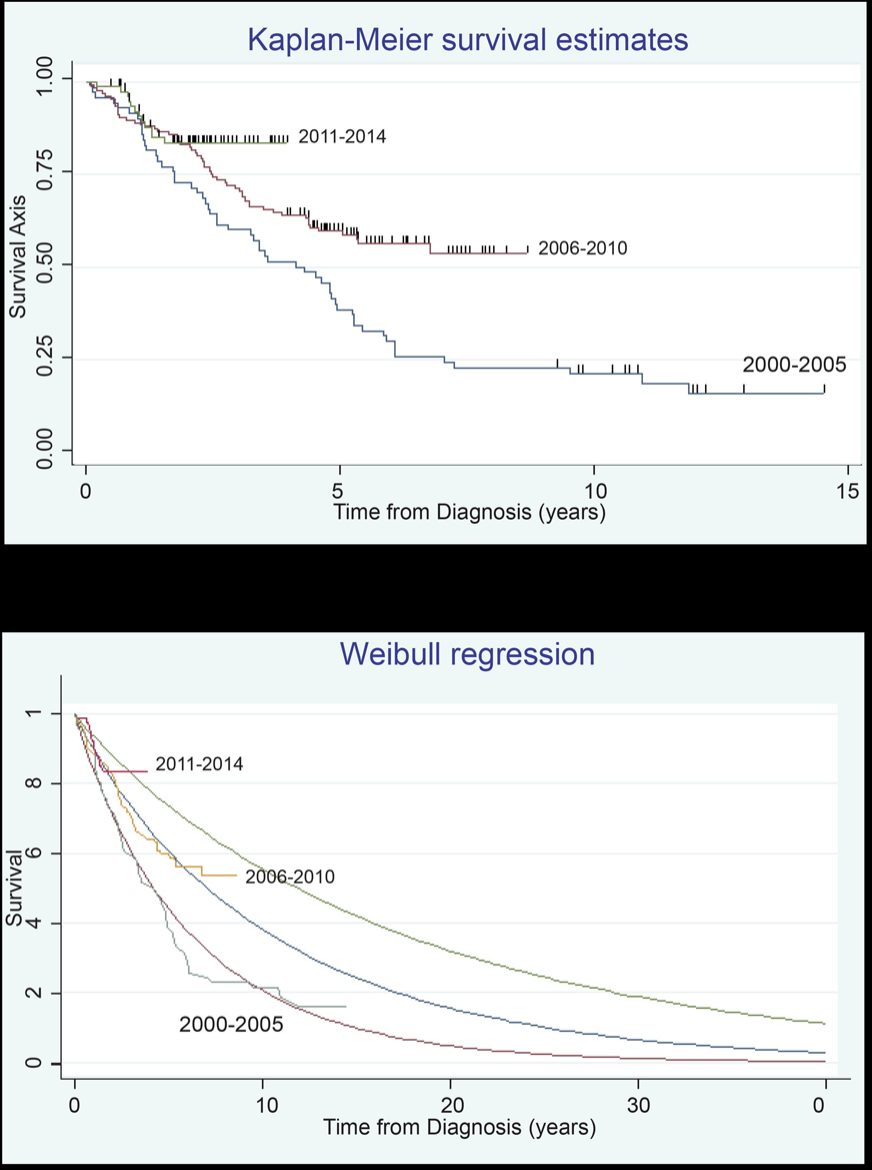
Survival of Multiple Myeloma patients in Austria overtime and extrapolated Weibull model survival estimates. The upper part (Fig 2A) illustrates the survival of Myeloma patients in Austria respective to the date of their first diagnosis in between 2000–2005 (blue line), 2006–2010 (red line), and 2011–2014 (green line). The x-axis represents time in years, and the y-axis represents the proportion of patients surviving. The dotted lines give the respective Weibull extrapolation of the patients’ 10 years survival. The lower part (Fig 2B) illustrates the survival of Myeloma patients in Austria respective to the date of their first diagnosis in between 2000–2005 (blue line), 2006–2010 (red line), and 2011–2014 (green line). The x-axis represents time in years, and the y-axis represents the proportion of patients surviving. The dotted lines give the respective Weibull extrapolation of the patients’ 10 years survival.

To benchmark Austrian treatment results to the international therapeutic landscape as reported in the SEER results [1] we stratified patients according to first diagnosis intervals in between the year 2000 to 2005 (70 patients., 13 [18.6%] alive), 2006 to 2010 (137 patients., 81 [59.1%] alive), 2011 to 2014 (83 patients, 71 [85.5%] alive) and before the year 2000 (20 patients., 2 [10%] alive).

An actual median survival (range) was observable for the groups of patients diagnosed before the year 2000 at 9.38 years (7.03–11.72) and for those patients, diagnosed in between 2000 and 2005 at 5.06 years (3.95–6.17), while the cohorts diagnosed 2006–2010 and from 2011 onwards have not yet reached their respective median survival. To better understand the future prognosis of these different patient cohorts a Weibull regression analysis was performed. The projected 10-year survival for the subgroups estimated via a Weibull [12] model, were approximately 21% (diagnosis 2000–2005), 39% (diagnosis 2006–2010) and 56% (diagnosis 2011–2014).

### Survival of MM patients in Austria from start of 3^rd^ line therapy (Fig 3)

Median survival after initiation of 3^rd^ line therapy was found to be 27 months (95% CI, 24.25–34.16 months). Maximum (ongoing) survival was found to be 170 months (0–170 months). 70 (67%) of the patients were deceased at the time of analysis and 35 (33%) were still alive. The proportion of patients surviving longer than 2, 3, 4 and 5 years was found to 70%, 64%, 47%, and 30% respectively.

**Fig 3:**
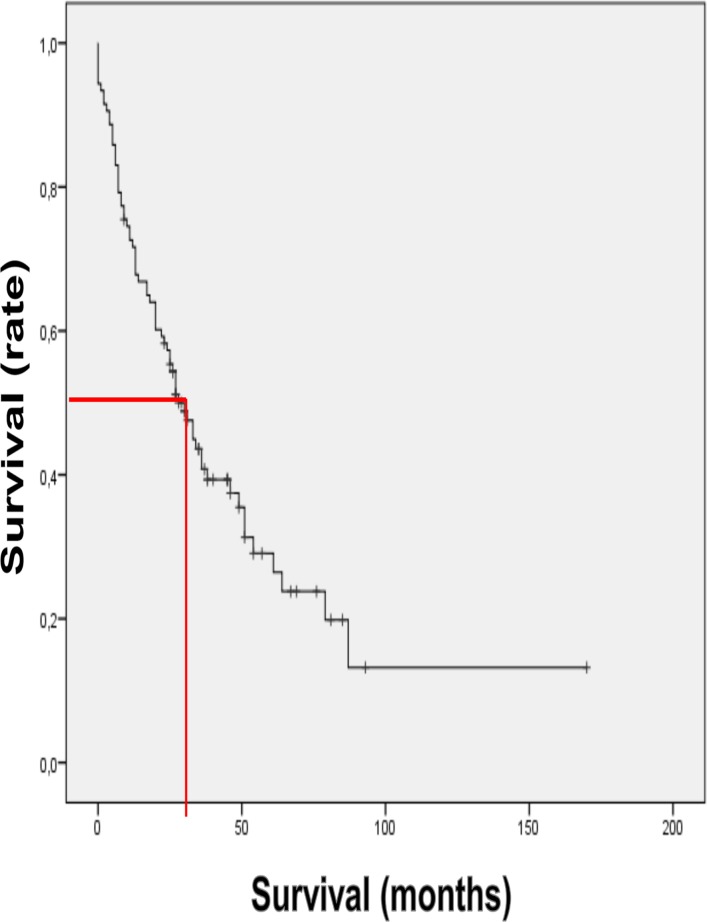
Survival of Multiple Myeloma patients in Austria from start of 3^rd^ line. **Therapy.** illustrates the survival of Myeloma patients in Austria from start of 3^rd^ line therapy in 105 patients documented in AMR median survival was found to be 27 months (0–170 months/ongoing).

## Austrian Treatment Patterns in 3^rd^ Line Therapy and Beyond

25 stem cell transplantations (SCTs) were used as a salvage modality in 19 (18.1%) patients. These transplants were instituted in a 3^rd^ line setting in 13 (12.4%) patients, 4^th^ line in 9 (8.6%) patients and in 5^th^ line or later in 3 (2.9%) patients. Transplants were autologous in 17 (16.2%) cases and allogeneic in 8 (7.6%) cases SCTs were part of the treatment sequences in all patients surviving over 5 years. Third line therapies were monotherapies in 18%, doublets in 43%, triplets in 36% and other combinations in 3% of cases. 4^th^ line and later therapies were monotherapies in 24%, doublets in 56%, triplets in 13% and other combinations in 7% of cases.

Regimens used in 3^rd^ line therapies (105 therapies) contained (adds up to > 100% due to combination therapies): steroids (80 treatments, 76.2%), BTZ (40, 38.1%), experimental agents (EA) (35, 33.3%), LEN (27, 25.7%), classic cytostatic agents (CCA) (15, 14.3%), THAL (8, 7.6%) and bendamustine (BEN) (7, 6.7%).

Regimes used in 4^th^ line therapies (68 therapies) contained (adds up to > 100% due to combination therapies): steroids (30, 44.1%), BTZ (26, 38.2%), BEN (14, 20.6%), EA (8, 11.8%), CCA (8, 11.8%), LEN (3, 4.4%), and THAL (2, 2.9%).

Median TNT between 3^rd^ and 4^th^ line therapies (TNT^3-4^) was 29.5 days (52 patients) and 31 days (21 patients) between 4^th^ and 5^th^ line therapies (TNT^4-5^). Excluding primary refractory patients by IMWG definition (PD under therapy or within 60 days from end of therapy)^8^ TNT^3-4^ was 221 days (21 patients) and TNT^4-5^ was found to be 197 days (9 patients.).

## Discussion

This analysis of Austrian myeloma patients’ treatment regimens and outcomes as documented in the AMR confirms the worldwide trend [1] of improving overall survival in this clinical entity over the last decade. The improvement based on the implementation of the first generation of novel agents (BTZ, THAL, LEN) into clinical routine increased median survival to 5.1 years in patients diagnosed between the years 2000 to 2005, and to 5.5 years in patients diagnosed between 2006 to 2010. This compares well with a median OS of 4.6 years for the 2000–2005 cohort and a 6.1 year median OS for the 2006–2010 subgroup reported by Kumar [1] based on the SEER data. In this study there was a small subgroup of patients (20 pts.) diagnosed before the year 2000, but documented in the AMR (initiated ~Jan 1 2008) because they were still alive 8 years later, also showing an excellent median survival of 9.4 years (95% CI, 7.0 to 11.7 years), but very probably this represents a major selection bias as myeloma patients surviving that long, in times of restricted therapeutic options, obviously belonged to a highly favorable biological subgroup. Furthermore, this group of pts. had the unique advantage to be eligible for new highly active *“novel agent”* based salvage regimes, when these entered clinical practice after 2005. As the number of diagnoses made before the registry was put in practice (2008) cannot be known exactly no conclusion should be drawn from the performance of this small subgroup of patients, but on the predominantly prospectively collected data from the subsequent patient cohorts, we think to be much more informative.

Using a Weibull model, we were able to extrapolate 10-year survival probabilities The 10-year survival probability of patients diagnosed between 2011 and 2014 was 56%, which is considered an improved survival rate in an unselected cohort of myeloma patients that has not been seen in previous years. We want to stress the fact that this represents “real-world” evidence collected in a representative, multi-center setting in routine patients, rather than in a highly selected clinical trial population. The continuous improvement in MM survival is accompanied by changing treatment paradigms. From 2005 onwards the use of BTZ and the IMiDs is significantly increasing, while after 2011 their simultaneous use in combination therapies could be observed ever more frequently (see Tables 1 and 2). It seems highly probable that this treatment pattern changes are the basis of the therapeutic progress made in the observation period.

The use of autologous transplantation (ASCT) remained more or less constant at ~ 35% of patients in the 1^st^ line setting over the whole period, comparing well to international practice patterns [13], while the use of 2^nd^ line ASCT increased from 5.5% to 18.7% of patients (Table 2). The median age of this myeloma cohort was 63.86 +/- 11.82 years at first diagnosis thus roughly half of them would not qualify for an ASCT using a threshold of 65 years of age widely in use in Europe. As 31% of pts. (22% in 1^st^ line, 9% in 2^nd^ line) received an autologus SCT early and another 25 SCTs were performed in later lines more than half of the eligible patients were actually transplanted. Furthermore, the detailed analysis of transplant use over time shows an increase of combined 1^st^ and 2^nd^ line ASCT rates from 35.6% (200–2005), to 50.7% (2006–2010) and finally 56.1% (2011–2014).

This is well in line (actually better) with a recent multinational analysis from 22 countries showing a transplant use of 48% in eligible patients over all lines [14].

In the USA it is well known that ASCT is even more heavily underused and a National Cancer Data Base analysis of [15] 137.409 MM patients diagnosed 1998–2010 found an ASCT rate in 1^st^ line of 12.1% only for the year 2010. In summary ASCT is not comparatively underused in our MM population but quite on the contrary has been applied extensively.

In addition, we were able to show promising results in advanced disease outcomes, as defined by start of 3^rd^ line therapy. Median survival from this therapeutic checkpoint was 27 months (0–170 months-ongoing), even without using 2^nd^ generation drugs (POM, CFZ), with a sizeable proportion of patients (about~ 20%) surviving over 5 years. This corroborates the thesis of Bart Barlogie [16] claiming that we already achieve functional cure of Myeloma in a subset of patients. All patients in this “*plateau*” cohort have received some type of stem cell therapy as part of their salvage regimes, highlighting the importance not to neglect the option of late salvage transplantation, even in times of multiple novel agents being available.

Treatments applied in the AMR setting in the 3^rd^ and 4^th^ line plus setting where dominated by combination regimes with a more intensive pattern in 3^rd^ line (more triplets) than in 4^th^ line were therapeutics were mostly used as palliative care. In responding patients TNT in both 3^rd^ and 4^th^ line or higher was ~ 200 days, but patients in this settings should not be taken off therapy for prolonged periods if possible.

The patterns of drug use correspond to the national registration status, guidelines [17] and practice with 1^st^ line therapy predominantly BTZ based, 2^nd^ line predominantly LEN based. BTZ-IMiD combinations and experimental agents (33.3% in 3^rd^ line, 11.8% in 4^th^ line) dominated 3^rd^ line combinations. In 4^th^ line, BEN and other cytostatic drugs were frequently combined.

The surprisingly overall survival results reported for 3^rd^ line myeloma therapy may, in part, also been influenced by the existence of a subgroup of patients (especially in the 2000–2005 cohort), who did not receive highly effective novel agent based 1^st^ line and/or 2^nd^ line therapies as BTZ, became available in Europe not until May 2005 and LEN has only recently (FEB, 2015) been registered for 1^st^ line use by EMA. These patients may have been more easier to salvage than optimally pretreated patients, although we know that (e.g. long term data from the VISTA trial [18]) the maximum benefit of an optimal induction therapy can never be recovered by a later introduction of effective drugs [19].

The MM-003 trial [3] led to the registration of POM as 3^rd^ line therapy standard, as well as the ASPIRE trial [20] will possibly doing the same thing for CFZ in Europe. MM-003 reported an median OS of 12.7 months with POM-DEX in a randomized phase III setting (with a high dose dexamethasone control arm) and very heavily pretreated pts., while ASPIRE reported a median progression free survival of 26.3 months exploring a combination of CFZ, LEN and DEX (KRd) with the median OS not reached at the time of publication, and Rd being the comparator, in a stricter 3^rd^ defined line setting. The seemingly inferior results of MM-003 to our result of a median OS of 27 months in the AMR cohort, probably is due to the much more stringent inclusion criteria of this study aiming at true double refractory patients. Furthermore, the median number of therapies applied before POM-DEX in the MM03 trial was 5, making it de facto a 6^th^ line study excluding a direct comparison to our 3^rd^ line analysis. On the other hand ASPIRE once more confirms that the relatively best results in Myeloma therapy in suitable patients are achieved with high efficient combinations combining the most active drugs.

Despite the principle limitations of observational data, the small numbers in some subgroups, the non-random assignment of treatments applied on the basis of physicians discretion and patients preferences, we think the results noteworthy as the lack of any significant difference in treatment patterns or risk factor distribution between patients in need of advanced therapy lines and those that did not receive such interventions strongly argues against any inherent bias in the selection of patients in the AMR.

With data provided in this paper we have set a background on which the now ongoing implementation of further new anti-myeloma drugs in routine care can be monitored for comparative clinical effectiveness. Furthermore, the ongoing linkage of AMR data to Myeloma biobanking and quality of life research will allow to address more refined questions like biomarker identification or questions in Health Technology Assessment (HTA).

With the upcoming emergence of additional active anti-myeloma compounds, the perspective to further improve on these results seems bright and perspectives of poor survival in patients with advanced myeloma should be regarded as a thing of the past.

## Supporting Information

S1 File S1 File lists all of AMR centers & coordinating physicians. (DOC)

## References

[1] Kumar SK, Dispenzieri A, Lacy MQ, Gertz MA, Buadi FK, Pandey S, et al Continued improvement in survival in multiple myeloma: changes in early mortality and outcomes in older patients. Leukemia. 2014; 28(5):1122–8. 10.1038/leu.2013.313 24157580 PMC4000285

[2] Kumar SK, Lee JH, Lahuerta JJ, Morgan G, Richardson PG, Crowley J, et al Risk of progression and survival in multiple myeloma relapsing after therapy with IMiDs and bortezomib: A multicenter international myeloma working group study. Leukemia 2012; 26:149–157. 10.1038/leu.2011.196 21799510 PMC4109061

[3] San Miguel J, Weisel K, Moreau P, Lacy M, Song K, Delforge M, et al Pomalidomide plus low-dose dexamethasone versus high-dose dexamethasone alone for patients with relapsed and refractory multiple myeloma (MM-003): a randomised, open-label, phase 3 trial. The Lancet Oncol. 2013;14: 1055–1066. 10.1016/S1470-2045(13)70380-2 24007748

[4] Ocio EM, Richardson PG, Rajkumar SV. Palumbo A, Mateos MV, Orlowski R, et al New drugs and novel mechanisms of action in multiple myeloma in 2013: a report from the International Myeloma Working Group (IMWG). Leukemia. 2014; 28: 525–42. 10.1038/leu.2013.350 24253022 PMC4143389

[5] Siegel DS, Martin T, Wang M, Vij R, Jakubowiak AJ, Lonial S, et al A phase 2 study of single-agent carfilzomib (PX-171-003-A1) in patients with relapsed and refractory multiple myeloma. Blood. 2012 10 4;120(14):2817–25. 10.1182/blood-2012-05-425934 Epub 2012 Jul 25. 22833546 PMC4123387

[6] Singal A, Higgins PDR, Waljee AK. A Primer on Effectiveness and Efficacy Trials. Clinical and Translational Gastroenterology (2014) 5, e45; 10.1038/ctg.2013.13 24384867 PMC3912314

[7] FDA website. Available: http://www.fdanews.com/CTA0617151?hittrk=CTA1561&utm_source=Real%20Magnet&utm_medium=Email&utm_campaign=76759603

[8] Coordinating ethical review board: Ethic commission of the Medical University of Innsbruck Innrain 43, A-6020 Innsbruck, Austria. Decision: vote 3252, session266/ 4.2, passed JUL 10, 2008

[9] Rajkumar SV, Harousseau JL, Durie B, Anderson KC, Dimopoulos M, Kyle R, et al Consensus recommendations for the uniform reporting of clinical trials: report of the International Myeloma Workshop Consensus Panel 1. Blood 2011;117: 4691–5. 10.1182/blood-2010-10-299487 21292775 PMC3710442

[10] Kaplan EL; Meier P (1958) "Nonparametric estimation from incomplete observations". J. Amer. Statist. Assn.53 (282): 457–481. JSTOR 2281868.

[11] Chng WJ, Dispenzieri A, Chim CS, Fonsecca R, Goldschmidt H, Lentzsch S, et al IMWG consensus on risk stratification in multiple myeloma. Leukemia 2014;28: 269–77. 10.1038/leu.2013.247 23974982

[12] Collett D. Modelling Survival Data in Medical Research. 1997: Chapman & Hall: London.

[13] Mohty M, Terpos E, Mateos MV, Palumbo A, Lejniece S, Beksac M, et al Frontline therapy for multiple myeloma (MM) in real-world clinical practice: Results from the third interim analysis of the multinational, non-interventional, observational EMMOS study. IMW 2015, Rome, #087.

[14] Mohty M, Terpos E, Mateos MV, Palumbo A, Lejniece S, et al. Analysis of Final Data from the Multinational, Non-Interventional, Observational Emmos Study (NCT01241396) in Patients (Pts) with Multiple Myeloma (MM) in Real-World Clinical Practice). Accepted for ASH 2015 (# 3034)

[15] Al-Hamadi MA, Shahrukh SK, Go RS. Use of autologous hematopoietic cell transplantation as initial therapy in multiple myeloma and the impact of socio-geo-demographic factors in the era of novel agents. American Journal of Hematology, Vol. 89, No. 8, 8 2014: 825–830. 10.1002/ajh.23753 24799343

[16] Barlogie B, Mitchell A, van Rhee F, Epstein J, Morgan GJ, Crowley J. Curing myeloma at last: defining criteria and providing the evidence. Blood 2014;124: 3043–51. 10.1182/blood-2014-07-552059 25293776 PMC4231416

[17] Gunsilius E, Drach J, for the Myeloma Platform of the Austrian Society of Hematology and Oncology (ÖGHO). memo 2010;3: 7–10.

[18] San Miguel JF, Schlag R, Khuageva NK, Dimopoulos MA, Shpilberg O, Kropff M, et al Continued Overall Survival Benefit After 5 Years' Follow-up with Bortezomib-Melphalan-Prednisone (VMP) Versus Melphalan-Prednisone (MP) in Patients with Previously Untreated Multiple Myeloma, and No Increased Risk of Second Primary Malignancies: Final Results of the Phase 3 VISTA Trial. Blood (ASH Annual Meeting Abstracts) 2011 118: Abstract 476.

[19] Palumbo A, Bringhen S, Ludwig H, Dimopoulos MA, Bladé J, Mateos MV, et al Personalized therapy in Multiple Myeloma according to patient age and vulnerability: a report from the European Myeloma Network (EMN). Blood 2011;118: 4519–29. 10.1182/blood-2011-06-358812 21841166

[20] Stewart AK, Rajkumar SV, Dimopoulos MA, Masszi T, Ŝpička I, Oriol A, et al for the ASPIRE investigators. Carfilzomib, lenalidomide, and Dexamethasone for Relapsed Multiple Myeloma. NEJM 2014; 10.1056/NEJMoa1411321

