# Supplementary material for: Real-World Use of 3rd Line Therapy for Multiple Myeloma in Austria: An Austrian Myeloma Registry (AMR) Analysis of the Therapeutic Landscape and Clinical Outcomes prior to the Use of Next Generation Myeloma Therapeutics
Source: PLoS One. 2016 Mar 3;11(3):e0147381. doi: 10.1371/journal.pone.0147381 (PMC4777410; doi:10.1371/journal.pone.0147381)
Supplement: S1 File — (DOC) [file pone.0147381.s001.doc]

**Appendix A**

**Centers and Contributors to the Austrian Myeloma Registry (AMR)**

*Patronage – Austrian Society for Hematology & Oncology (ÖGHO)*

***Austrian Centers Local Coordinators, Physicians***

County Hospital Lienz, Austria Dr. med. Alois Walder

County Hospital Hall in Tirol, Austria Dr. med. Horst Oexle

County Hospital Reutte, Austria Dr. med. Stephan Schreieck

County Hospital St. Johann, Austria Dr. med. Michael Schnallinger

Hospital of the Elisabethan Order, Linz, Austria Dr. med. Hedwig Kasparu

Dr. med. Daniel Lechner

Hospital St. Vincenz, Zams, Austria Prof. Dr. med. Ewald Wöll

Outpatient Clinic: Innsbruck, Austria Prof. Dr. med. Christof Ludescher

“Ambulatorium Hämatologie & Onkologie” Dr.med. Johanna Kantner

University Hospital Innsbruck, Austria Prof. Dr. med. Eberhard Gunsilius

Dr. med. Ella Willenbacher

Dr. med. Wolfgang Willenbacher

Mag. Roman Weger

Wihelminenspital, Vienna, Austria Prof. Dr. med. Heinz Ludwig Prof. Dr. med. Niklas Zojer

State Hospital Vorarlberg, Feldkirch, Austria Dr.med. Alois Lang

Hanusch Hospital, Vienna, Austria Prof. Dr. Felix Keil

Dr. med. Adelheid Seebacher

***International Centers Local Coordinators, Physicians***

Szent István and Szent László Hospital, Budapest, Prof. Dr. med. Tamás Maszi

Hungary

Università degli Studi di Bari, Bari, Italy Prof. Dr. med. Domenico Ribatti

Dr. med. Christian Marinaccio
